# Investigating the effects of synbiotic supplementation on functional movement, strength and muscle health in older Australians: a study protocol for a double-blind, randomized, placebo-controlled trial

**DOI:** 10.1186/s13063-024-08130-9

**Published:** 2024-05-07

**Authors:** David J. Barry, Joshua B. Farragher, Andrew C. Betik, Jackson J. Fyfe, Lilia Convit, Matthew B. Cooke

**Affiliations:** 1 School of Health Sciences, Swinburne University of Technology, Melbourne, VIC Australia; 2 Institute for Physical Activity and Nutrition (IPAN), School of Exercise and Nutrition Sciences, Deakin University, Geelong, VIC Australia; 3 Centre for Sports Research (CSR), School of Exercise and Nutrition Sciences, Deakin University, Burwood, VIC Australia; 4 Sport, Performance and Nutrition Research Group, School of Allied Health, Human Services and Sport, La Trobe University, Bundoora, VIC Australia

**Keywords:** Synbiotic, Microbiome, Sarcopenia, Muscle mass, Muscle strength, Physical performance, Gut-muscle axis

## Abstract

**Background:**

Aging has been associated with a progressive loss of skeletal muscle quality, quantity and strength, which may result in a condition known as sarcopenia, leading to a decline in physical performance, loss of independence and reduced quality of life. While the cause of impaired physical functioning observed in elderly populations appears to be multifactorial, recent evidence suggests that age-associated alterations in gut microbiota could be a contributing factor. The primary objective will be to assess the effects of a dietary synbiotic formulation on sarcopenia-related functional outcomes such as handgrip strength, gait speed and physical performance within older individuals living independently. The secondary objective will be to examine associations between changes in gut microbiota composition, functional performance and lean muscle mass.

**Methods:**

Seventy-four elderly (60–85 years) participants will be randomized in a double-blind, placebo-controlled fashion to either an intervention or control group. The intervention group (*n* = 37) will receive oral synbiotic formulation daily for 16 weeks. The control group (*n* = 37) will receive placebo. Assessments of physical performance (including Short Physical Performance Battery, handgrip strength and timed up-and-go tests) and muscle ultrasonography will be performed at 4 time points (baseline and weeks 8, 16 and 20). Likewise, body composition via bioelectric impedance analysis and blood and stool samples will be collected at each time point. Dual-energy X-ray absorptiometry will be performed at baseline and week 16. The primary outcomes will be between-group changes in physical performance from baseline to 16 weeks. Secondary outcomes include changes in body composition, muscle mass and architecture, fecal microbiota composition and diversity, and fecal and plasma metabolomics.

**Discussion:**

Gut-modulating supplements appear to be effective in modifying gut microbiota composition in healthy older adults. However, it is unclear whether these changes translate into functional and/or health improvements. In the present study, we will investigate the effects of a synbiotic formulation on measures of physical performance, strength and muscle health in healthy older populations.

**Trial registration:**

This study was prospectively registered with the Australian New Zealand Clinical Trials Registry (ACTRN12622000652774) in May 2022.

**Supplementary Information:**

The online version contains supplementary material available at 10.1186/s13063-024-08130-9.

## Background

The global demographic is experiencing a shift to an older population with increased life expectancy. As we age, we observe a progressive loss of muscle mass, strength, and physical function which can have many health implications, a condition known as sarcopenia. The term, *sarcopenia*, was proposed by Irwin Rosenberg to describe the age-related decline in skeletal muscle and physical performance [1]. The reported prevalence of sarcopenia in the literature appears to vary due to different criteria for diagnosis [2, 3] and heterogeneity of study populations [4]. A recent meta-analysis estimated a global prevalence of sarcopenia in older adults (≥ 60 years) between 10 and 27% [5], with another suggesting an overall prevalence of 10% in the general population [6]. The latter findings are similar to those of a 2020 meta-analysis by Papadopoulou and colleagues which reported prevalence rates of 9 and 11% in community-dwelling women and men, respectively [7]. Diminished muscle strength has been more closely tied to impaired physical function and poor outcomes than reduced muscle mass in the revised European Working Group on Sarcopenia in Older People (EWGSOP2) consensus definition of sarcopenia [8]. EWGSOP2 [8] criteria propose a diagnosis of sarcopenia is *probable* with low muscle strength (i.e., reduced grip strength) and this diagnosis is *confirmed* with the identification of low muscle mass (quantity). Sarcopenia is considered *severe* if reduced physical performance (i.e., slow gait speed) is identified in conjunction with reduced skeletal muscle strength and mass according to these revised EWGSOP2 criteria.

While the cause of age-related changes in muscle mass and physical functioning is likely multifactorial, recent evidence suggests that alteration in gut microbiota may play a role [9, 10]. It has been estimated that the human gastrointestinal tract (GIT) contains approximately 10^14^ microorganisms with more than 1000 distinct bacterial species [11]. While *Bacteroidetes*, *Firmicutes*, *Proteobacteria*, and *Actinobacteria* are the predominant bacterial phyla represented in the gut microbiota [12], each person (host) appears to have a unique biological relationship with its microbiota [13]. Analyzing fecal samples obtained from 728 older women, Jackson et al. found a negative association between host frailty and microbiota diversity [14]. The gut-muscle axis has received attention recently with suggestions that the composition and diversity of gut microbiota can be a determinant of skeletal muscle mass and metabolism [9]. Emerging evidence, predominantly in pre-clinical models, suggests disruption to the balance between microbial communities, such as depletion of symbionts and commensal bacteria and over-representation of opportunistic pathogens (such as those seen in gut dysbiosis), may contribute to the aforementioned age-related changes [9, 15, 16]. Indeed, with advancing age, where malnutrition, inactivity and chronic diseases are often experienced, gut dysbiosis is observed [17]. Conversely, individuals with successful aging, such as centenarians, demonstrate elevated representation of “good” bacteria, such as *Bifidobacteria* [18].

Few studies have explored the gut-muscle axis in humans, especially within the elderly. Bacterial taxa such as *Enterobacteriaceae*, *Bacteroides*, and *Prevotella*, have been correlated with measures of gait speed [15]. A 2022 study found that the diversity and composition of gut microbiota in community-dwelling older adults was associated with sarcopenia [19]. Claesson et al. demonstrated that physical performance was inversely related to species richness of the fecal microbiota of 178 older subjects [20]. Analysis of data from 371 ELDERMET cohort subjects revealed an association between the presence of frailty in community-dwelling elderly individuals and comparable gut microbiome profiles of nursing home residents [21]. Furthermore, an adequate representation of *Bifidobacterium* has shown to be fundamental for the production of butyrate [22], a short-chain fatty acid (SCFA) with important anti-inflammatory and pro-anabolic activities involved in the gut-muscle axis. Treatment with butyrate appears to protect against age-related muscle atrophy [23], which supports the idea that pre- and/or probiotic supplementation, known to increase the abundance of *Bifidobacterium* and butyrate producers in older individuals [24, 25], may protect against physical performance deficits with aging [16]. Despite this, there is a scarcity of research evaluating the effect of gut-modulating supplements in older populations. A recent systematic review found no clear evidence of benefit in physical performance in 5 studies evaluating the use of pre-, pro-, or synbiotics in older adults [26]. Notwithstanding, 12 weeks of consuming a synbiotic increased SCFA production compared with a placebo in elderly individuals in a 2013 study [27]. In a more recent trial, 13 weeks of consuming a prebiotic improved handgrip strength compared with placebo [28] which suggests the potential for prebiotic supplementation as a treatment for deficits in age-related muscle function. Similarly, ingestion of a multi-strain probiotic for 12 weeks resulted in significant improvements in physical performance (timed up and go (TUG) test, gait speed) and decreased risk of falls compared with placebo [29].

Skeletal muscle mass and strength loss and functional impairment with advancing age are linked to several chronic diseases and associated with a higher chance of falls/fractures [30], loss of independence [31] and the likelihood of requiring long−term care due to reduced mobility, disability, and/or hospitalization [32, 33]. Since older adults are the fastest−growing global subpopulation, modulation of the microbiome through dietary administration of pre−, pro−, or synbiotics may represent a promising approach to counterbalance the loss of muscle mass and function seen with aging [34]. This manuscript describes the evaluation of synbiotic supplementation on physical performance measures compared with placebo according to the Standard Protocol Items: Recommendations for Intervention Trials (SPIRIT) reporting guidelines [35].

## Methods

### Study design

We will carry out a randomized, placebo-controlled, double-blinded superiority study to evaluate the effects of a synbiotic formulation on measures of physical performance, strength and muscle health in older, independent living individuals. This clinical trial was registered with the Australian New Zealand Clinical Trials Registry (ACTRN12622000652774) in May 2022 and the study design, protocol, and informed consent procedures were approved by the Human Research Ethics Committee of Swinburne University of Technology (Ref. 20226246–9780).

### Study setting

**Fig. 1:**
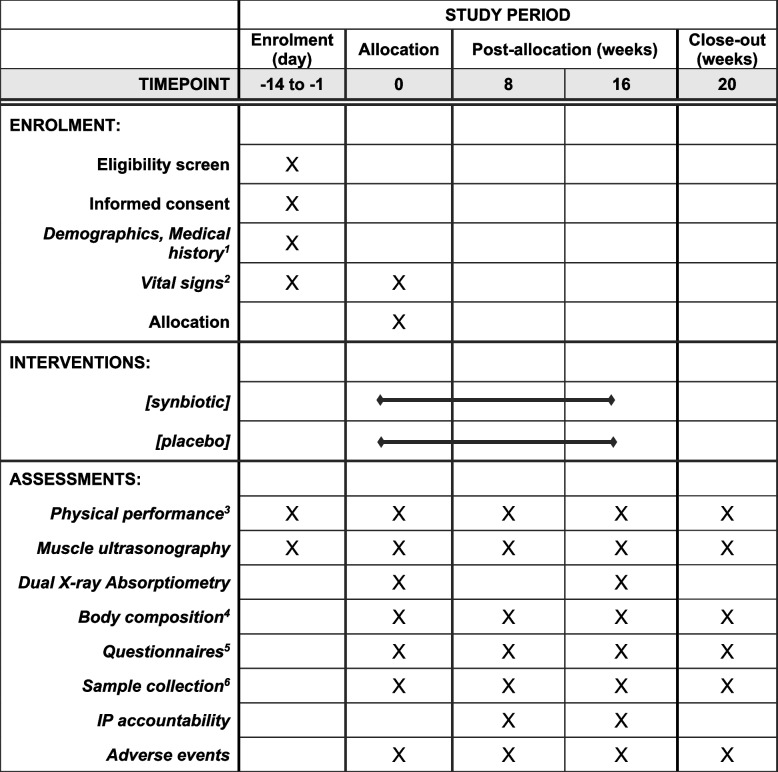
Schedule of enrolment, interventions, and assessments (adopted from SPIRIT 2013 Figure). ^1^include age, gender, COVID-19 history and vaccine status; ^2^include heart rate, and blood pressure; ^3^include handgrip strength, 4m gait speed, balance testing, repeat chair stands, timed up and go; ^4^via bioelectric impedance analysis; ^5^include SARC-F, Physical Activity Scale for the Elderly, Gastrointestinal Symptom Rating Scale, Constipation Assessment Scale; ^6^include stool sample and blood sample. IP: investigational product

This clinical trial will take place at Swinburne University of Technology, Hawthorn campus. Dual-energy X-ray absorptiometry (DXA) evaluations will be conducted at Deakin University, Burwood campus. Recruitment commenced in November 2022 and the enrolment target is expected to be met in April 2024. The schedule of enrolment, interventions, and assessments is shown in Fig. 1.

#### Study intervention

All eligible participants will be randomized equally (1:1) into either treatment (synbiotic) or control (placebo) groups. The study treatment will consist of a multi-strain synbiotic (SYN) powder containing inulin and a combination of probiotic strains. Placebo (PLA) will consist of maltodextrin, which will match synbiotic in appearance, taste, and consistency. Both SYN and PLA sachets will be 1800 mg. The participants will be instructed to consume 1 sachet daily (orally, dissolved in water, and at the same time each day) for the entire intervention period. Both SYN and matching PLA will be provided by Lallemand Health Solutions (Montreal, QC, Canada). Enrolled participants will be asked to make no changes to their current exercise habits or usual dietary intakes and abstain from taking any dietary supplements containing prebiotics and/or probiotics during the study. A list of prohibited medications is provided in Supplementary File 1.

#### Study duration

**Fig. 2:**
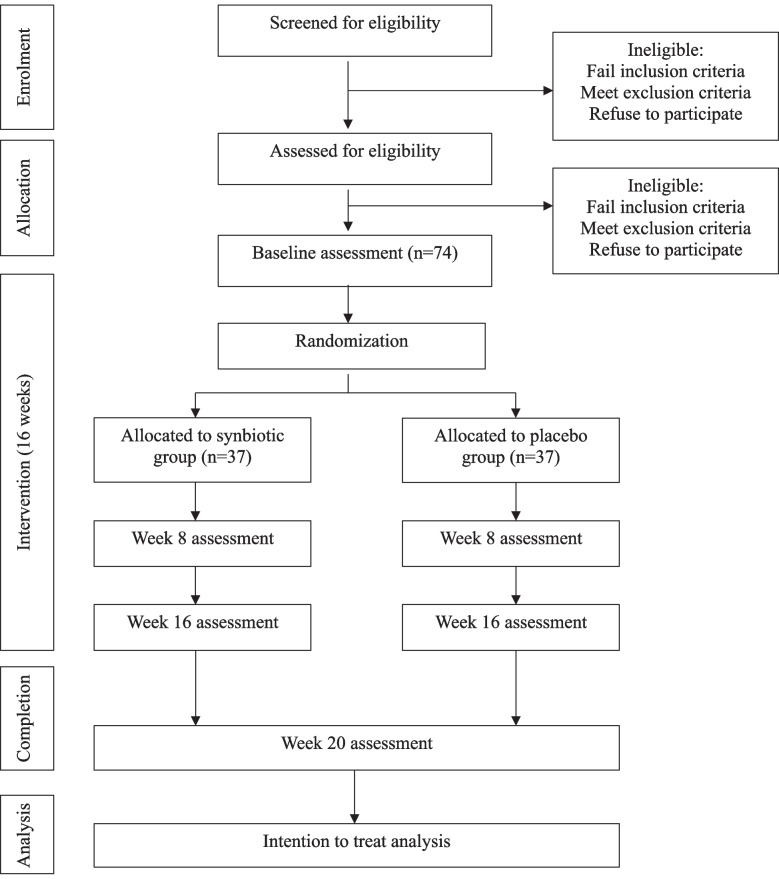
Study flow chart

Participant involvement will be approximately 22 weeks. This will consist of a screening period of no longer than 2 weeks, a 16-week intervention period, and a 4-week follow-up period. Each participant will attend a screening visit, during which eligibility will be confirmed and informed consent will be obtained. Study investigational product (IP) will be initiated within 2 weeks following successful screening (at baseline visit). Each participant will subsequently attend 3 further visits: mid-intervention at 8 weeks, an end-of-intervention visit at 16 weeks, and a follow-up visit at 20 weeks. Study endpoints will be evaluated at each visit as discussed below. The trial design is outlined in Fig. 2.

#### Study participants

Approximately 74 participants will be enrolled to receive oral synbiotic formulation or placebo (*n* = 37 per group), daily for 16 weeks. Community-dwelling male and female participants between 60 and 85 years of age (inclusive) will be enrolled.

### Eligibility

#### Inclusion criteria

To be eligible for enrolment into the study, participants must meet all of the following:

1. Evidence of a personally signed and dated information consent form (ICF) document indicating that the participant has been informed of all pertinent aspects of the study.
2. Healthy adults* who are determined by medical history and clinical judgment of the investigator to be eligible for inclusion in the study.

*Note: Healthy participants with pre-existing stable disease, defined as disease not requiring significant change in therapy or hospitalization for worsening disease within 6 weeks before enrolment, can be included.

(3) Male and nonchildbearing-potential female adults between 60 and 85 years of age (inclusive) at the time of enrolment (signing of the ICF).

Female participants of nonchildbearing potential must meet at least 1 of the following criteria:

- Postmenopausal status, defined as follows: cessation of regular menses for at least 12 consecutive months with no pathological or physiological cause;
- Have undergone a documented hysterectomy and/or bilateral oophorectomy;
- Have medically confirmed ovarian failure.

All other female participants are considered to be of childbearing potential.

(4) Willing and able to comply with scheduled visits, laboratory tests, and other study procedures.
(5) Willing to refrain from consuming probiotic and/or prebiotic supplements as well as foods that contain naturally occurring probiotics (e.g., fermented foods with live, active cultures such as yogurt, kefir, kombucha, kimchi, curd, buttermilk) from enrolment (Screening) until the end of the study.
(6) Able to walk 10 m.
(7) Able to get up from a chair.
(8) BMI between 18 and 30 (inclusive) and body weight of at least 40 kg.

#### Exclusion criteria

Participants with any of the following characteristics/conditions will not be included in the study:

1. Use of probiotics, prebiotics or antibiotics in the past 4 weeks (screened participants that are otherwise eligible may enrol into the study after a 4-week wash-out period).
2. Use of proton pump inhibitors in the last 3 months.
3. Chronic treatment with statins or other drugs with known myotoxicity.
4. Musculoskeletal or other disorder resulting in inability to perform physical function testing.
5. Presence of medical conditions causing secondary sarcopenia.
6. Presence of diseases or disorders that can impact muscle mass.
7. At risk for malnutrition (Mini Nutritional Assessment-Short Form [MNA-SF] ≤ 7).
8. Diagnosed gastrointestinal disease with known association with gut microbiota dysbiosis.
9. Lower or upper extremity fracture within the past 6 months and/or hip replacement surgery within the past 12 months.
10. Myocardial infarction in the past 6 months.
11. Coronary artery disease, peripheral vascular disease, previous stroke, or history of transient ischemic attacks.
12. Uncontrolled hypertension (> 160/100 mmHg).
13. Androgen therapy in males or estrogen therapy in females.
14. Kidney failure.
15. History of cholecystectomy.
16. Blindness.
17. Inability to read or understand English.
18. Unintentional weight loss of 5% or more in the last 30 days or 10% or more in the last 6 months.
19. Known infection with human immunodeficiency virus, hepatitis B virus, or hepatitis C virus.
20. Known or suspected immunodeficiency, as determined by history and/or laboratory/physical examination.
21. Treatment with immunosuppressive therapy, including cytotoxic agents or systemic corticosteroids.
22. Other acute or chronic medical or psychiatric condition that, in the judgment of the investigator, would make the subject inappropriate for entry into this study.
23. Any condition or abnormality that, in the judgment of the investigator, would compromise the safety of the participant or the quality of the study data.
24. Currently enrolled in another clinical study or having participated in another clinical study in the 30 days before the screening visit.
25. Milk or soy allergy.

### Randomization and masking

Participants who fulfil the eligibility criteria and consent to participate will be randomly assigned (1:1) to receive either a synbiotic (intervention) or placebo (control). Randomization will be completed by an independent (unblinded) third party using a random number generator in Microsoft Excel. To perform random allocation, stratified block randomization will be used. The unblinded staff will have no interaction with study participants and will not be involved in the collection or analysis of data. They will assign each participant to one of two intervention groups (synbiotic or placebo) and allocate IP based on stratification factors consisting of gender, age, and handgrip strength (“normal” or “low”, based on EWGSOP2 criteria [8]; men: “normal” ≥ 27kg or “low” < 27 kg and women: “normal” ≥ 16kg or “low” < 16 kg). Stratification data will be collected by blinded study investigators from each participant during their screening visit and will be submitted to the unblinded staff responsible for group allocations.

### Allocation concealment and masking

Intervention and placebo will be matched in appearance, taste and consistency, and will have the same packaging and weight. The unblinded staff will be provided with the code for determining which containers are treatment or control and will label all IP containers for both intervention groups. All study participants and investigative site staff involved in data collection will be blinded to treatment allocation.

### Implementation

Screening, enrolment, confirming eligibility, obtaining consent, and all other data collection and analysis will be performed by investigative site staff who are blinded to treatment allocation. Randomization and treatment allocation will be performed by unblinded study staff who will have no other study involvement.

### Objectives

#### Primary aim

To determine the effect of a synbiotic formulation on indicators of functional performance, balance, and muscle strength.

#### Secondary aim

To determine the effect of a synbiotic formulation on muscle mass and architecture, microbiota composition and diversity and their associated metabolites, and self-reported indicators of sarcopenia.

#### Safety aim

To assess the safety and tolerability of a synbiotic formulation in older individuals.

### Study assessments

Data collection will include Short Physical Performance Battery (SPPB), handgrip strength (HGS), timed up and go (TUG) test, as well as body composition and muscle morphology. These outcomes will be assessed at 4 time points (baseline and weeks 8, 16, and 20). Venous blood will be collected at 4 time points by venipuncture of the median cubital vein after overnight fasting using commercial collection tubes. Two 4.5-mL samples will be taken in EDTA and two 8.5mL SST collection tubes. Samples will be rested for 30 min at room temperature followed by centrifugation at 3000 × *g* for 15 min at 4°C after which serum and plasma will be collected into 0.5mL aliquots and stored at − 80°C until analysis. Participants will be carefully instructed on the procedures for fecal sample collection. Participants will be given stool collection kits at each visit, to be collected at home and returned at the following visit, as described below.

### Primary outcome measure: SPPB

Physical performance will be measured with the SPPB, which is a well-validated and widely used tool [36] that has excellent test–retest reliability and predictive validity [37, 38]. The SPPB measures three components (i) standing balance, (ii) 4-m habitual gait speed, and (iii) repeated chair rises. Each of the three domain scores ranges from 0 to 4 points, yielding a composite score ranging from 0 to 12 points. Higher scores indicate better function. A total score of fewer than 10 points indicates a high risk of frailty and falls [38] and a one-point change is considered clinically meaningful [39, 40]. SPPB component measures will be scored according to Table 1.

**Table 1:** Scoring schema for SPPB

**Scoring schema for standing balance**	
**Balance Tests**	**Balance score**
Side-by-side stand	a) Not held for 10 s = 0 points
	b) Held for 10 s = 1 point
Semi-tandem stand	a) Not held for 10 s = 0 points
	b) Held for 10 s = 1 point
Tandem stand	a) Less than 3 s = 0 points
	b) Between 3 and 9.99 s = 1 point
	c) Held for 10 s = 2 points
**Scoring schema for 4 m gait speed**	
Unable or > 60 s	0 points
< 60 s but > 8.70 s	1 point
6.21 s to 8.70 s	2 points
4.82 s to 6.20 s	3 points
< 4.82 s	4 points
**Scoring schema for repeated chair rises**	
Unable or > 60 s	0 points
< 60 s but > 16.69 s	1 point
13.70 s to 16.69 s	2 points
11.20 s to 13.69 s	3 points
< 11.20 s	4 points

For the *balance tests*, participants will be asked to stand unassisted in 3 positions:

1. Side-by-side stand (feet in a side-by-side position);
2. Semi-tandem stand (heel of one foot is beside the big toe of the other foot);
3. Tandem stand (toe of one foot is behind and touching the heel of the other foot).

Participants will be asked to maintain each position for 10 s. If unable, the time achieved will be recorded and the next position will be attempted.

*Gait speed* will be measured as the time taken to walk 4 m (between markers set at 3 and 7 m of a 10 m walking course) at the participant’s usual speed. Assistive devices will be allowed if needed. Gait speed will be calculated as meters per second (m/s) and trained staff will record time to the nearest 0.01 s. The best (fastest) of the two attempts will be included in data analysis. Gait speed is comparable to the full battery for predicting disability [38], and slower gait speeds predict higher rates of frailty and hospitalization [41]. Coleman et al. determined 0.11 m s^−1^ to be a clinically meaningful change [42].

For *repeated chair rises*, participants will be asked to rise from an armless chair, sit back down, and repeat this movement five times as quickly as possible without using their arms [43]. Participants will begin the test with their arms folded across their chest, sitting in a chair with a seat height of 43 cm. Participants will be instructed to stand up completely (defined as an upright trunk with full knee and hip extension), make firm contact when sitting and keep their arms folded across their chest. Time (in seconds) will be recorded, beginning upon the prompt, “go,” and stopping when the participant’s buttocks returned to the seat following the fifth stand [44]. No words of encouragement will be used. Chair stand time has been described as a reliable [45], useful measure of lower leg strength [43], fall risk [46], and balance control [47], and has been shown to complement measures of gait speed when screening for sarcopenia [48].

### Primary outcome measure: handgrip strength

Handgrip strength will be measured using a Jamar analog hand dynamometer (Patterson Medical, Warrenville, IL) with the handle set to position II. Participants will be instructed to grip the device and squeeze as hard as possible with their dominant hand [49]. Three trials will be recorded with participants in a sitting position, elbow by their side and flexed at 90°, forearm and wrist in a neutral position, with shoulder adducted and neutrally rotated, as previously described [50]. The maximum of these values will be used for analyses [51]. Grip strength has been shown to predict future function [52] and has been negatively associated with frailty [53].

### Primary outcome measure: TUG test

The TUG test measures the time (in seconds) taken to stand up from a standard chair, walk to and around a marker placed 3 m away at a comfortable pace, return to the chair and sit back down [54]. Participants will be permitted to use walking aids (if necessary) and will be instructed not to use their arms to stand up. Beginning with the participant seated with their back against the backrest of the chair, timing will commence on the command “go” and will stop when the participant returns fully seated in the back of the chair. The task will be performed twice, with the faster (shorter) time used in data analysis. TUG has been used to examine gait, agility, fall risk, balance, and dynamic (turning) movements in older adults [43, 55–57].

### Secondary outcome measure: body composition

Dual-energy X-ray absorptiometry (DXA) will be used to measure whole and regional body composition, including lean body mass (LBM, g), appendicular lean mass (ALM, g), total and regional body fat mass (FM, %), and fat-free mass (FFM, g). Fasting whole-body scans (GE Healthcare Lunar iDXA, Madison, WI) will be acquired with participants in a supine position on the scanner table, wearing light clothing, with their limbs close to their bodies according to the manufacturer’s protocol. Segmental analyses of the whole body into arm, leg, and trunk segments will be separated with anatomical landmarks by the DXA analysis software. Appendicular skeletal muscle mass (ASMM) will be calculated as the sum of the lean soft tissue mass (LSTM) of both the right and left extremities, with the assumption that all non-bone and non-fat tissue is skeletal muscle [58]. Skeletal mass index (SMI) will be determined as the ALM divided by height squared (kg/m^2^) [59]. Body composition via DXA will be evaluated at baseline and week 16.

Body mass index (BMI), total body and segmental fat percent and weight, total body water percent and weight, total body and segmental muscle mass, and FFM will be estimated using bioelectric impedance analysis (BIA) with a multi-frequency body composition analyzer (Tanita MC-780U, Tokyo, JP) at 4 time points. Impedance will be analyzed in a fasting state, with participants standing barefoot on the electrode platforms, holding grips with both arms straight down at their sides.

### Secondary outcome measure: muscle morphology

Muscle mass and architecture will be quantified via ultrasound examination (Sonosite iViz, Fujifilm, Tokyo, JP) of the *rectus femoris* (RF) and *vastus intermedius* (VI) at 4 time points. Participants will lie supine on an examination table, hips in a neutral position, knees in full extension, ankles at 90°, and both feet on the table, as previously described [60]. The assessed muscles will be in a relaxed state (5 min) to avoid muscle contraction-induced fluid shifts [61], and scans will be obtained before any functional testing [62]. A horizontal reference line will be drawn at 50% of the distance between the greater trochanter and the superior border of the patella of the dominant leg. These anatomical landmarks were chosen based upon guidelines proposed by the SARCopenia through UltraSound (SARCUS) working group of the European Geriatric Medicine Society [60, 63]. With adequate transmission gel applied to the transducer head, two images will be obtained at this point: (i) an image in the transverse plane with the transducer placed perpendicular to the long axis of the thigh, and (ii) an image in the sagittal plane with the transducer placed parallel to the long axis. All images obtained will be subsequently analyzed using ImageJ (NIH) software [64]. Muscle thickness (MT), echo intensity (EI), cross-sectional area (CSA), and subcutaneous tissue thickness (STT) will be measured on the transverse images; fascicle length (FL) and pennation angle (PA) will be measured on the sagittal images [65, 66]. Measurements will be taken three times, and the mean value will be recorded and included in the data analysis [67]. Low levels of muscle mass as determined by the ratio of fascicle length to muscle thickness will be factored to obtain the Ultrasound Sarcopenia Index (USI) [68].

### Secondary outcome measure: fecal microbiota composition and diversity

Stool samples will be collected at 4 time points by participants at home within 72 h of each of their upcoming study appointments. Spontaneously voided feces will be collected into specimen containers, placed within provided biohazard bags, and immediately frozen until transfer to our research site, where they will be stored at − 80 °C until analysis. DNA extraction and 16S rRNA or whole-genome shotgun (WGS) sequencing will be performed to determine α- and β-diversity and taxonomic abundance [69, 70].

### Secondary outcome measure: anthropometric variables

At baseline, body mass will be recorded in light clothing to the nearest 0.1 kg using a digital scale (Tanita, Tokyo, JP). Height (without shoes) will be measured using a stadiometer (SECA 213, Hamburg, Germany). BMI will be calculated as weight (kg) divided by height (m)^2^.

### Secondary outcome measure: calf circumference

A non-elastic tape measure (SECA 201, Hamburg, Germany) will be used to measure calf circumference (CC) at the widest part of the non-dominant leg with participants in a supine position. The tape will be placed flat on the skin, taking care not to compress the sub-dermal tissue. Studies support the utility of CC as a proxy marker for measuring muscle mass [71–73]. CC has shown moderate to high sensitivity and specificity in predicting sarcopenia [74, 75] and predicts disability risk in older adults [76]. CC will be assessed at baseline and week 16.

### Secondary outcome measure: dietary assessment

Habitual dietary intake will be recorded by participants using 3-day dietary intakes for the days leading up to each of the 4 study appointments. Dietary intake data will be analyzed to discern energy intake, and macro- and micro-nutrients. Participants will also complete the Australian Eating Survey [77] food frequency questionnaire at baseline only and before randomization to establish pre-intervention dietary patterns.

### Secondary outcome measure: SARC-F

SARC-F is a recently developed rapid screening tool for sarcopenia [78]. It is a self-reported questionnaire consisting of 5 components: Strength, Assistance with walking, Rising from a chair, Climbing stairs and Falls, respectively. Each of the components is scored from 0 to 2 points, yielding a composite score ranging from 0 to 10 points. Lower scores indicate better function. A total score of ≥ 4 points is indicative of sarcopenia. A recent meta-analysis [79] reported that SARC-F has excellent specificity in screening for sarcopenia, however, due to its low to moderate sensitivity [80], it may detect only severe cases [8]. Participants will complete SARC-F questionnaires at each study visit.

### Safety outcome measures

Serum and plasma samples will be analyzed by an external commercial pathology facility to determine changes (outside normal variation) in clinical chemistry (Comprehensive Metabolic Panel [CMP]) and clinical hematology markers (Complete Blood Count [CBC]) elicited after baseline and through week 16. Adverse events (AEs) and serious adverse events (SAEs) will be assessed and recorded from baseline through week 20.

### Hypotheses and study endpoints

#### Primary aims

We hypothesize 16 weeks of ingesting a synbiotic preparation compared to placebo will result in statistically significant improvements (within-group and between-group) in:

1. 4-m gait speed
2. Standing balance
3. Repeated chair rises
4. Timed up and go
5. Handgrip strength

#### Secondary aims

We hypothesize 16 weeks of ingesting a synbiotic preparation compared to placebo will result in statistically significant improvements (within-group and between-group) in:

(6) Muscle mass and body composition (LBM, ALM, total and regional body FM and FFM)
(7) Muscle quality (CSA, MT, EI, FL, and PA of *Rectus femoris* and *Vastus intermedius* muscles)
(8) Fecal microbiota composition and diversity
(9) Untargeted fecal and serum metabolomics

#### Safety aims

We hypothesize synbiotic administration will be safe and well-tolerated as defined by the absence of:

(10) Changes (outside normal variation) in CBC elicited after baseline and through week 16
(11) Changes (outside normal variation) in CMP elicited after baseline and through week 16
(12) AEs elicited after baseline and through week 20
(13) SAEs until study completion

### Statistical analyses and power

This estimated sample size for the main outcome measures of physical performance was based on an assumed correlation of 0.7 between the pre- and post-intervention outcome measures, and an effect size of Cohen’s *d* = 1.2 for gait speed based on data from Román et al. [29] that examined the effects of a multi-strain probiotic on gait speed and the TUG test in a study cohort of 35 (17 and 18 in treatment and placebo groups, respectively). To have a power of 80% and a significance level of 5%, our study aims to enrol an estimated sample size of 74 participants (37 per group), taking into account a conservative ~ 30% dropout rate.

The study hypotheses will be addressed using a hierarchical linear model analysis to test for significant differences between the two comparative groups (intervention and placebo) over time. A primary intention to treat (ITT) analysis will be conducted on all the participants who complete the trial. Additionally, a secondary analysis will be carried out to include only participants who consumed ≥ 90% of their supplement over the course of the intervention period. Between-group comparisons will be made using linear models and statistical significance set at *p* < 0.05. Where there are significant group xtime interactions, planned contrasts will compare changes from baseline under each intervention. Similar tests will be performed for the secondary hypotheses, using appropriate transformations if these measures exhibit non-normal distributions across time points. This analysis will allow for the control of any baseline group differences and to test for moderation effects in terms of these variables over time. Analysis of microbial community composition will be conducted using Phyloseq and vegan package in R version 3.6.1 as per protocol.

Wilcoxon’s signed-rank test, Spearman’s rank correlation (p), Pearson’s correlation (r), multiple linear regression, and/or linear mixed models will be used to examine associations and appropriate comparisons depending on the distributions of the variables. Relationships between markers of functional performance, ordinations of stool microbiota composition, and stool/plasma metabolites will also be assessed using the Structural Equation Model to enable causal assumptions to be interpreted between variables to ensure these relationships are not “coincidental” and linear mixed models. All mixed models will include the subject as a random factor and time as a continuous covariate. Backward stepwise regression will be used to identify the strongest predictors of changes in aspects of functional performance. The false discovery rate for all tests will be controlled by adjusting *P* values using the Benjamini–Hochberg procedure. Adjusted *P* values will be presented as *Q* values. Statistical analyses will be completed using SPSS v.26. 11.2.2

### Risk–benefit analysis

All participants will be monitored and questioned for side effects. Both probiotics and prebiotics are safe for the majority of the population, but side effects can occur. Side effects of probiotics and prebiotics are usually minor and consist of self-limited gastrointestinal symptoms, such as a temporary increase in gas, mild abdominal discomfort, bloating, diarrhea, and constipation. Side effects can vary from mild to very serious and may vary from person to person. In some cases, these effects might be long-lasting, or permanent, and may even be life-threatening.

Food allergy symptoms (such as tingling or itching in the mouth; hives; swelling of the lips, tongue or other body parts; abdominal pain; diarrhea, or lightheadedness) may be experienced within a few minutes to 2 h after consuming the probiotic and prebiotic supplement if the participant is allergic to any of its ingredients.

Synbiotics (products containing a mixture of probiotics and prebiotics) are generally considered safe, particularly in healthy people; however, there are some risks. These risks are increased in participants who have compromised immune systems or have other serious medical conditions. Possible harmful effects can include developing infections or antibiotic resistance, or the production of harmful by-products from microorganisms contained in the synbiotic.

The risks of adverse events associated with this study are minimal, and any adverse events will be recorded and managed following Good Clinical Practice (GCP) guidelines and legal requirements. However, this study will provide important outcomes regarding the potential benefits of synbiotic consumption on physical function, strength and body composition, which may provide supporting evidence for the use of synbiotics as a cost-effective supplement for older individuals. Participants will be fully informed about the risks associated with the study before providing informed consent and will have the right to withdraw at any time. This will be explained in the ICF document and verbally during the screening process.

### Safety reporting

To ensure ongoing participant safety, at each visit, the research team will ask about any symptoms they have developed or any changes to their health that may have resulted from the IP. An adverse event (AE) will be defined as any untoward medical occurrence in a participant administered IP that does not necessarily have a causal relationship with the intervention [81]. An AE can be any unfavorable and unintended sign (including an abnormal laboratory finding), symptom or disease occurring, which appears to be associated with the administration of the IP. All AEs and serious adverse events (SAEs) that are deemed related to a participant’s involvement in this study will be collected, recorded, and reported to the authorizing ethics committee, following local requirements. A SAE will be defined as an adverse event that:

- Is life-threatening (places the subject at immediate risk of death).
- Requires in-patient hospitalization or prolongation of existing hospitalization.
- Results in persistent or significant disability/incapacity.
- Results in death.

### Relationship to study intervention

The principal investigator (PI) will assess all AEs and SAEs to determine whether there is any potential relationship to the study intervention. The relationship will be assessed as either “related” or “unrelated” as below:

- *Related*: There is a reasonable possibility that the study intervention caused the AE or SAE, or there appears to be a temporal relationship between the administration of the study intervention and the event.
- *Unrelated*: There does not seem to be a reasonable possibility that the administration of the study intervention caused the AE or SAE, there does not appear to be a temporal relationship between the study intervention and event onset, or there has been an alternate etiology established that explains the event.

### AE and SAE reporting

All AEs and SAEs will be captured on the *adverse events log*.

The PI will be responsible for notifying the approving HREC of any SAE, independent of the relatedness to the study, within 7 calendar days upon initial receipt of the information. If the SAE is considered as related to the IP, a report must follow within 7 days of learning about the SAE.

### Period for collecting AEs/SAEs

The period for collecting AEs and SAEs (“active collection period”) for each participant is as follows:

- AEs: From informed consent until the end of study involvement (week 20).
- SAEs: From informed consent until study completion.

### Participant withdrawal and/or discontinuation from the study

Participants will be informed they may withdraw from the study at any time. This will be explained during the consent process and is detailed in the ICF. Participants will be made aware they may be discontinued at any time at the discretion of the investigators for safety reasons, or the inability of the participant to comply with the protocol-required schedule of study visits or procedures. Investigators may discontinue or withdraw a participant from the study for the following reasons:

- Non-compliance to study protocol.
- Occurrence of AE related to the intervention, laboratory abnormality, or other medical condition such that participation in the study would not be in the best interest of the participant. This includes whether the participant meets an exclusion criterion (newly developed or not previously recognized).
- Disease progression requiring discontinuation of the study intervention.

Throughout the duration of the study, the research team will evaluate the well-being of all participants. If continued participation is considered harmful to an individual’s health or safety, that participant will be withdrawn from the study. If a participant is discontinued or withdraws from the study, every attempt will be made to have them complete an Early Termination Visit to determine the participant outcome, to ensure they are withdrawn safely, and to close off any unresolved AEs, if possible. Participants who withdraw after randomization will not be replaced.

### Ancillary and post-trial care

The study team will ensure proper and safe conduct of the trial and will strive to mitigate risks to participants associated with their involvement. Due to the wide safety profile of the investigational product, we do not anticipate the need for compensation due to harm, and there are no provisions for ancillary or post-trial care. Likewise, we do not expect there to be cause for terminating the trial. However, in the event a participant is harmed as a direct consequence of their involvement, they will be referred to their treating health practitioner and directed to contact the Swinburne University Human Research Ethics Committee. This information is included in the consent forms and will be discussed at the screening appointment. There are no provisions for post-trial access to the intervention.

### Compliance with study intervention

Participants will be instructed to take their allocated study intervention once daily for 16 weeks. At week 8 (mid-intervention) and week 16 (end-of-intervention), participants will be asked to return their study IP (including unused supplements and empty packaging). IP accountability will be performed to confirm compliance with the required dosing schedule. In addition, participants will be asked to complete and return a study supplement adherence log, which will be reviewed in week 8 and 16 visits.

### Recruitment and retention

Local, community-dwelling individuals will be invited to participate. Recruitment will occur via various mailing lists, hard-copy flyers, and social media outlets. It is anticipated the majority of enrolment may be from participants of previous studies who have consented to future contact. See Supplementary File 2 for the Recruitment Plan. Once enrolled, retention strategies will include systematically scheduling future appointments, SMS appointment reminders and verbally confirming upcoming appointments at each visit.

### Data collection and management

#### Confidentiality of data

To maintain privacy, participants will be randomly assigned unique study identification numbers. Data collection forms (DCFs) will be developed before the onset of the trial. DCFs for each participant will be completed by a study investigator. All essential data (DCFs, source documents, signed consent documents and study logs) will be securely stored in a locked cabinet in the research site office accessible only to the PI and delegated research staff. De-identified study data will be collected and managed using research electronic data capture (REDCap) tools [82]. The PI and delegated research staff will have access to the final datasets.

#### Data collection

Investigators will be trained and delegated by the PI for all study requirements, including specimen collection, eliciting of information from participants in a uniform and reproducible manner, and the standardized approach to all study assessments described above. DXA scans will be conducted by trained, blinded staff who hold current certifications.

#### Data monitoring

The blinded research team will conduct ongoing monitoring of the study to confirm the safety and rights of all participants are protected, that the currently approved study protocol is being followed and to verify data collected is complete and accurate. This trial will be conducted in accordance with the ethical principles for medical research involving human subjects described in the Declaration of Helsinki [83] and consistent with the International Conference on Harmonisation Good Clinical Practice (ICH-GCP) and applicable regulations of Australia. The research team will perform internal quality control (QC) procedures to confirm source data and biological specimen collection and documentation are completed as per study protocol. Data QC checks will also be regularly generated within the REDCap database to ensure data entry is accurate and complete. This will be a small (*n* = 74), single-site RCT, and the blinded study team will manage and monitor trial data; as such no formal data monitoring committee (DMC) will be formed. Any protocol deviations (PDs) or violations (PVs) will be documented in the study records and reported in accordance with HREC requirements.

## Discussion

In this manuscript, we present a protocol to examine the effects of a synbiotic on physical performance in an older population. Gut dysbiosis has been associated with increasing biological age [84]. Evidence from animal and human studies supports the role of the gut microbiome in maintaining lean body mass and physical functioning [16].

A high percentage of healthcare costs in elderly populations arise from the negative outcomes resulting from reduced skeletal muscle mass and strength [85]. Sarcopenia has been shown to represent a significant but modifiable economic burden on healthcare services worldwide [86]. Goates et al. estimated the total cost of hospitalizations for individuals with sarcopenia was USD 40.4 billion in the USA alone [87]. Older adults are the fastest-growing global subpopulation and by 2025 the number of people with sarcopenia is predicted to increase to 1.2 billion [88]. Therefore, a better understanding of mechanisms that underlie the maintenance of skeletal muscle mass, strength, and physical performance may provide valuable insight to help address the public health priority of healthy aging. The gut microbiome may be involved in the maintenance of these outcomes. Existing evidence has focused on the diversity of the gut microbiota rather than the functional capacity of its microbiome. Studies exploring the evolving role of gut microbial changes resulting in the loss of skeletal muscle or with specific measures of physical function remain inconclusive. To our knowledge, no human studies have yet investigated utilizing synbiotic (pre- and probiotic combination) interventions to influence gut microbiota and the association with measures of physical performance in elderly, community-dwelling individuals.

Therefore, the current RCT aims to provide a greater understanding of the relationship between gut microbiota, its microbiome and measures of physical function and muscle health in older adults that may ultimately help in reducing the progression of sarcopenia and/or frailty. If proven effective, the outcomes of this project may provide further evidence to support the development of targeted interventions (i.e., synbiotics) to influence the microbiota composition leading to the maintenance of skeletal muscle mass and strength and improved physical performance in elderly populations.

Given the growing interest in the role of microbial populations in the aging gut, further research is needed to confirm and/or extend some of these initial findings. An improved understanding of the impact of the gut microbiome on skeletal muscle could have a dramatic effect on improving the health and quality of life for the elderly, reducing associated comorbidity and disability, and stabilizing rising healthcare costs. It is anticipated that the outcomes of this project may describe the safety and tolerability of therapeutic microbiome manipulations and further explore the utility of targeted interventions to maintain muscle mass and improve physical functioning in elderly populations.

### Trial status

This trial will be conducted according to the current ethics-approved Protocol (v1.2, 25 Jun 2022). Protocol amendments, defined as any modification to the study objectives, design, or procedures that impact participant safety or conduct of the study, will be approved by SUHREC before implementation in the trial. Any such protocol amendments will be communicated to all investigative site staff and will be updated on ANZCTR. These modifications will be communicated to participants at their next scheduled appointment and, if appropriate, will be reflected in revised SUHREC-approved ICF documents that will be discussed with all participants before obtaining re-consent. Supplementary File 3 provides the protocol amendment history. Recruitment for this study commenced in November 2022 and the enrolment target is expected to be met in April 2024.

### Universal trial number

U1111-1277–2798

### Supplementary Information

**Additional file 1.** Clarification tables for inclusion and exclusion criteria

**Additional file 2.** Recruitment plan

**Additional file 3.** Protocol amendment history

## Data Availability

The datasets generated from this trial will be available from the corresponding author upon reasonable request.

## Abbreviations

AE: Adverse event
ALM: Appendicular lean mass
ANZCTR: Australian New Zealand Clinical Trials Registry
ASMM: Appendicular skeletal muscle mass
BIA: Bioelectric impedance analysis
BMI: Body mass index
CBC: Complete blood count
CC: Calf circumference
CFU: Colony-forming unit
CMP: Comprehensive metabolic panel
CSA: Cross-sectional area
DCF: Data collection form
DUHREC: Deakin University Human Research Ethics Committee
DXA: Dual-energy X-ray absorptiometry
EDTA: Ethylene diamine tetraacetic acid
EI: Echo intensity
EWGSOP: European Working Group on Sarcopenia in Older People
FFM: Fat-free mass
FL: Fascicle length
GCP: Good Clinical Practice
GIT: Gastrointestinal tract
HREC: Human Research Ethics Committee
ICF: Information and Consent Form
ICH-GCP: International Conference on Harmonisation- Good Clinical Practice
ICMJE: International Committee of Medical Journal Editors
IP: Investigational product
ITT: Intention to treat
LBM: Lean body mass
LSTM: Lean soft tissue mass
MNA-SF: Mini Nutritional Assessment- Short Form
MT: Muscle thickness
NIH: National Institutes of Health
PA: Pennation angle
PI: Principal investigator
QC: Quality control
RCT: Randomized controlled trial
RF: *Rectus femoris*
rRNA: ribosomal RNA
SAE: Serious adverse event
SARCUS: SARCopenia through UltraSound
SCFA: Short-chain fatty acid
SMI: Skeletal mass index
SPIRIT: Standard Protocol Items Recommendations for Interventional Trials
SPPB: Short Physical Performance Battery
SST: Serum separator tube
SUHREC: Swinburne University Human Research Ethics Committee
TUG: Timed up-and-go
USI: Ultrasound sarcopenia index
VI: *Vastus intermedius*
WGS: Whole-genome sequencing
