# Supplementary material for: Investigating the effects of synbiotic supplementation on functional movement, strength and muscle health in older Australians: a study protocol for a double-blind, randomized, placebo-controlled trial
Source: Trials. 2024 May 7;25:307. doi: 10.1186/s13063-024-08130-9 (PMC11077830; doi:10.1186/s13063-024-08130-9)
Supplement: Supplementary file 1 — Additional file 1. Clarification tables for inclusion and exclusion criteria [file 13063_2024_8130_MOESM1_ESM.docx]

CLARIFICATION TABLES FOR INCLUSION AND EXCLUSION CRITERIA

| **MEDICATIONS** | |
| --- | --- |
| EXCLUDED | INCLUDED |
| Antibiotics‡ | Pain medications – analgesics |
| Statins (if participant has commenced or the dose has changed in the past 3 months) | Statins (if dose has remained stable for past 3 months or more) |
| Antimalarials | Antacids |
| Colchicine | Warfarin |
| Zidovudine | Herbal remedies |
| Proton pump inhibitors |  |
| Steroids (glucocorticoids) |  |
| Laxatives |  |
| Cytotoxic or immunosuppressive agents |  |
|  |  |
| **NUTRACEUTICALS** |  |
| EXCLUDED | INCLUDED |
| Probiotics supplements‡ | Herbal remedies |
| Prebiotics supplements‡ | Vitamin and mineral supplements |
| Fiber supplements† | Fish oil |
| ‡Screened participants that are otherwise eligible may enrol into the study after a 4-week wash-out period. | |
| †Fiber supplements refer to sources of functional dietary fiber used for the purpose of attaining beneficial physiological effects on the gut (i.e. to promote regular bowel movements). Fiber supplements can include products designed and marketed specifically for bowel health, such as Metamucil, as well other non-branded dietary fiber sources such as psyllium husks or oat bran. | |
