# Supplementary material for: Investigating the effects of synbiotic supplementation on functional movement, strength and muscle health in older Australians: a study protocol for a double-blind, randomized, placebo-controlled trial
Source: Trials. 2024 May 7;25:307. doi: 10.1186/s13063-024-08130-9 (PMC11077830; doi:10.1186/s13063-024-08130-9)
Supplement: Supplementary file 2 — Additional file 2. Recruitment plan [file 13063_2024_8130_MOESM2_ESM.docx]

RECRUITMENT PLAN

Recruitment will occur via various mailing lists, hard copy flyers and social media outlets and may include the following:

- The Centre for Human Psychopharmacology at Swinburne University participant database who have consented to be notified of further studies
- Social media (Facebook)
- Advertisements via local businesses, retirement villages, senior centers, organizations, community groups/clubs (with permission)
- Swinburne University portal and email group distribution lists
- Media advertisements/advertorials in local newspapers and/or radio

**Swinburne University portal information**

*Investigating the effects of synbiotic supplementation on functional movement, strength and muscle health in older Australians.*

We are looking for men and women to participate in a study evaluating the effects of a synbiotic formulation (combination of a prebiotic and probiotic) on measures of physical performance, strength and muscle health in older populations.

**Background**

Our group is interested in characterising the bacterial communities that live in our gut (gut microbiome) and how they contribute to human health. Like many countries, Australia has a population that is rapidly ageing due to low birth rates and longer life expectancy. The proportion of the population aged > 65 years is predicted to rise markedly over the next two decades, which will impose significant social and economic challenges on the country. Previous studies indicate that there is a link between gut microbiome and physical function and so we aim to study this area in more detail in an older population.

**About this study**

The aim of this study is to determine the effectiveness of a synbiotic formulation on indicators of functional performance, balance and muscle strength. We are also hoping to evaluate the effectiveness of this formulation on muscle mass and quality, gut microbiota composition and diversity.

**Study criteria**

You may be eligible for this study if you:

- Are aged 60-85 years (inclusive).
- Have a BMI between 18 and 30 (inclusive) and body weight of at least 40 kgs.
- Are able to attend 5 appointments over a 22-week period at Swinburne University (Hawthorn campus)
- Currently have no disease that can affect normal healthy gut function.

Participants who complete the 22-week study will be provided a Coles Myer gift card.

**Participate in the study**

If you have any queries about this study or would like further information please call *9214 5560* or email [gutmuscle@swin.edu.au](mailto:gutmuscle@swin.edu.au)

This study has been approved by the Swinburne University Human Research Ethics Committee.

**Email template to previous study participants**

Dear [ENTER NAME],

Dr Matthew Cooke (Chief Investigator) from Swinburne University would like to invite you to participate in a study to test whether a synbiotic formulation (combination of a prebiotic and probiotic) improves physical performance, strength and muscle health in older populations compared to a placebo.

The aim of this study is to determine the effectiveness of a synbiotic formulation on indicators of functional performance, balance and muscle strength. We are also hoping to evaluate the effectiveness of this formulation on muscle mass and quality, gut microbiota composition and diversity.

You may be eligible for this study if you:

- Are aged 60-85 years (inclusive).
- Have a BMI between 18 and 30 (inclusive) and body weight of at least 40 kgs.
- Are able to attend 5 appointments over a 22-week period at Swinburne University (Hawthorn campus)
- Currently have no disease that can affect normal healthy gut function.

**Additional eligibility criteria apply**

Participants who complete the 22-week study will be provided a Coles Myer gift card.

Please [click here](https://www.swinburne.edu.au/research/centres-groups-clinics/centre-for-human-psychopharmacology/our-clinical-trials/) to visit our website for further information on the study.

We are currently recruiting for this study. If you are interested in registering to participate in this study or would like more information, please respond to this email, or contact:

Dr Matthew Cooke (Chief Investigator) or

Dr David Barry (Project Manager)

Ph: +61 3 9214 5560 | [gutmuscle@swin.edu.au](mailto:gutmuscle@swin.edu.au)

Thank you for your interest in Swinburne University’s research and for your willingness to participate as a volunteer in our trials. This study has been approved by the Swinburne University Human Research Ethics Committee.

**Facebook advertisement**

We are looking for men and women to participate in a study evaluating the effects of a synbiotic formulation (combination of a prebiotic and probiotic) on measures of physical performance, strength and muscle health in older populations.

*Click here* for more information or to participate
