# Supplementary material for: Investigating the effects of synbiotic supplementation on functional movement, strength and muscle health in older Australians: a study protocol for a double-blind, randomized, placebo-controlled trial
Source: Trials. 2024 May 7;25:307. doi: 10.1186/s13063-024-08130-9 (PMC11077830; doi:10.1186/s13063-024-08130-9)
Supplement: Supplementary file 3 — Additional file 3. Protocol amendment history [file 13063_2024_8130_MOESM3_ESM.docx]

PROTOCOL AMENDMENT HISTORY

| **Version** | **Date** | **Amendment Details** |
| --- | --- | --- |
| V0.2 | 28JAN21 | Updated inclusion and exclusion criteria, revised study procedures: Changes to better clarify study eligibility and visit procedures |
| V0.3 | 08AUG21 | Updated study procedures: Changes to study cohorts and procedures |
| V0.4 | 03JAN22 | Updated IP and study procedures: Changes to reflect finalization of strain selection, procedural clarification, addition of appendices 11.2, 11.3 and 11.4 |
| V0.5 | 11FEB22 | Protocol updates and study procedures: Protocol formatting changes, clarification of protocol deviations, addition of appendix 11.5 biological sampling |
| V1.0 | 23FEB22 | Referencing updates, revised visit procedures and timeframes, minor formatting changes |
| V1.1 | 23MAR22 | Revision of eligibility criteria, study procedures |
| V1.2 | 25JUN22 | Addition of COVID check, revised visit procedures and recruitment plan |
